# Comparative Transcriptome Analysis Reveals Heat-Responsive Genes in Chinese Cabbage (*Brassica rapa* ssp. chinensis)

**DOI:** 10.3389/fpls.2016.00939

**Published:** 2016-06-28

**Authors:** Aihua Wang, Jihong Hu, Xingxue Huang, Xia Li, Guolin Zhou, Zhixiang Yan

**Affiliations:** ^1^Wuhan Vegetable Research Institute, Wuhan Academy of Agricultural Science and TechnologyWuhan, China; ^2^State Key Laboratory of Hybrid Rice, College of life Sciences, Wuhan UniversityWuhan, China; ^3^Key Laboratory of Biology and Genetic Improvement of Oil Crops, Ministry of Agriculture, Oil Crops Research Institute of the Chinese Academy of Agricultural SciencesWuhan, China; ^4^BGI-ShenzhenShenzhen, China

**Keywords:** transcriptome, heat stress, RNA-seq, Chinese cabbage, NAC

## Abstract

Chinese cabbage *(Brassica rapa* ssp. chinensis) is an economically and agriculturally significant vegetable crop and is extensively cultivated throughout the world. Heat stress disturbs cellular homeostasis and causes visible growth inhibition of shoots and roots, severe retardation in growth and development, and even death. However, there are few studies on the transcriptome profiling of heat stress in non-heading Chinese cabbage. In this study, we investigated the transcript profiles of non-heading Chinese cabbage from heat-sensitive and heat-tolerant varieties “GHA” and “XK,” respectively, in response to high temperature using RNA sequencing (RNA seq). Approximately 625 genes were differentially expressed between the two varieties. The responsive genes can be divided into three phases along with the time of heat treatment: response to stimulus, programmed cell death and ribosome biogenesis. Differentially expressed genes (DEGs) were identified in the two varieties, including transcription factors (TFs), kinases/phosphatases, genes related to photosynthesis and effectors of homeostasis. Many TFs were involved in the heat stress response of Chinese cabbage, including NAC069 TF which was up-regulated at all the heat treatment stages. And their expression levels were also validated by quantitative real-time-PCR (qRT-PCR). These candidate genes will provide genetic resources for further improving the heat-tolerant characteristics in non-heading Chinese cabbage.

## Introduction

The species *Brassica rapa* includes various vegetable crops, such as turnip, field mustard, and Chinese cabbage. Non- heading Chinese cabbage (*B. rapa* ssp. chinensis) is an economically and agriculturally significant vegetable crop that is cultivated extensively worldwide. Non-heading Chinese cabbage originated from China and has a long cultivation history (Song et al., 2014a). The adaptable growth temperature for Chinese cabbage ranges from 18 to 22°C and its production is usually impaired by heat stress in many regions (Yu et al., 2012).

Heat stress, triggered by high environmental temperature, can affect plant performance, leading to severe retardation in vegetative growth, yield depression and even death (Caers et al., 1985; Barnabás et al., 2008; Song et al., 2014b). One of the phenotypes is leaf etiolation and bleaching with clear-cut inhibition of photosynthetic activity (Wang L. et al., 2011). Based on global climate model analysis, the predictions suggest that global warming and extreme heat events will threaten food safety by reducing crop production in the future (Battisti and Naylor, 2009; Rosenzweig et al., 2014). Therefore, discovery of the genes related to heat tolerance and investigating the molecular mechanism play important roles in genetic improvement of crops.

Signal transduction components, transcription factors (TFs) and proteins associated with the metabolism of stress-generated reactive oxygen species (ROS) are mainly responsive to the high temperature (Grover et al., 2013). Identification of heat responsive genes from suitable genotypes can give some insights into the heat-tolerance mechanism. Transcript profiling of two Chinese cabbage (*B. rapa* ssp. pekinensis) inbred lines showed that many genes are affected by high temperatures including heat shock proteins (HSPs), genes associated with membrane leakage and enzymes involved in ROS homeostasis (Dong et al., 2015). Previous studies have also shown that genes involved in oxidative stress, protection of proteins, programed cell death, biotic stress responses and metabolism were differentially expressed under high temperatures (Larkindale and Vierling, 2008).

In recent years, a great deal of attention has been paid to the elucidation of the mechanisms of heat-tolerance for breeding heat-resistant cultivars of Chinese cabbage and other important crops. In Chinese cabbage, heat-responsive miRNA and nat-siRNAs were identified, and some of these small RNA were upregulated under heat stress (Yu et al., 2012, 2013). Another non-coding small RNAs, chloroplast small RNAs (csRNAs), were also reported to be highly sensitive to heat stress and results showed that high temperature suppresses the production of some csRNAs (Wang L. et al., 2011). HSPs have also been assumed to play a central role in the heat stress response and in acquired thermotolerance in plants (Kotak et al., 2007). Using microarray, the transcript profiles of two Chinese cabbage inbred lines were studied and some heat responsive genes, such as heat shock proteins and MYB41, were identified (Dong et al., 2015). Lately, a total of 9687 novel lncRNAs were identified and 192 genes were regulated by these lncRNAs in Non-heading Chinese cabbage NHCC under heat treatment. However, the responsive genes to heat stress in non-head Chinese cabbage remain not fully understood.

Recently, the whole genome of *B. rapa* was sequenced and annotated, providing a solid foundation to study the expression of heat stress responsive genes (Wang X. W. et al., 2011). High-throughput sequencing methods such as Illumina SOLEXA, ABI SOLiD, and Roche 454 have observably increased the efficiency and reduced the cost of sequencing, making the study of transcriptomes easier and more feasible. RNA sequencing (RNA-Seq), a high-throughput sequencing method, is developed to analyze the transcriptome either with or without genome information. It's an efficient tool to promise simultaneous estimation of abundance and new transcript discovery (Cloonan et al., 2008). By RNA-Seq, researchers can obtain almost all of the expressed genes, especially genes with very low abundance. Therefore, genes with abundant expression differences and interesting pathways can be analyzed exhaustively. Compared with microarray, RNA-seq has very low, if any, background signal, and has also been shown to be highly accurate for quantifying expression levels (Wang et al., 2009). RNA-Seq has been applied for transcriptome analysis in many plant species under abiotic stress, including carnation (Wan et al., 2015), banana (Yang et al., 2015) and *Brassica juncea* (Bhardwaj et al., 2015).

In the present study, genome-wide analysis of gene expression in leaves of Chinese cabbage under heat stress was performed using RNA-seq based on the Illumina HiSeq2000 platform. The aim of the study is to identify heat responsive genes and provide new insight into the heat tolerance in non-heading Chinese cabbage.

## Materials and methods

### Plant materials

A heat sensitive variety “GHA” (S) and a tolerant variety “XK” (T) of non-heading Chinese cabbage (*B. rapa* ssp. chinensis) were used in this study. Sterile seeds were sown in pots and germinated in a growth chamber. For heat treatments, 3-week-old seedlings were grown at 37°C (high temperature) for 1, 6, 12, and 18 h. For control samples (CK), the leaf discs were incubated at 25°C. Then, leaves of the two varieties under CK and heat treatments were collected, frozen immediately in liquid nitrogen, and stored at −80°C for use.

### RNA isolation, cDNA library construction and illumina sequencing

Total RNA was isolated from the leaves using Trizol Reagent (Invitrogen, USA) according to the manufacturer's instructions. The extracted RNA was treated with DNase I (Promega, USA) to remove the contaminated DNA. The RNA quality and purity were varified by Nanodrop 2000 and electrophoresis on 1.0% agarose gels. Using poly-T oligo-attached magnetic beads, mRNAs were purified from the total RNA. Then, the mRNAs were fragmented and cDNA was synthesized using random hexamer, DNA polymerase I and RNase H. The double-stranded cDNAs were purified and ligated to adaptors for Illumina paired-end sequencing. The quality and quantity of the library was verified using an Agilent 2100 Bioanalyzer and ABI real time RT-PCR, respectively. The cDNA libraries were sequenced using the Illumina HiSeq2000 platform by the Beijing Genomics Institute (BGI).

### Sequence data analysis and annotation

Raw reads in fastq format were firstly filtered and the adaptor sequences and low quality reads were removed. Stringent filtering criterion was carried out to minimize the effects of sequencing errors during the assembly. Briefly, bases with Phred quality score lower than 20 and reads length short than 50 bp were discarded. Reads of 70% bases in one read having high quality scores (≥20) were filtered. The genome sequence of *B. rapa* retrieved from the Brassica Database (BRAD) (http://brassicadb.org/brad/) was used as the reference database (Wang X. W. et al., 2011). All the clean reads were then mapped to the reference genome using TopHat v 1.4.0 (Trapnell et al., 2010). The transcripts abundance was normalized by the FPKM (fragments per kilobase of exon per million fragments mapped) using Cufflinks (Trapnell et al., 2012).

### Identification of differentially expressed genes (DEGs)

The annotation of differentially expressed genes (DEGs) was performed by analyzing syntenic relationships between *B. rapa* and *Arabidopsis thaliana*. The syntenic orthologs of DEGs in *A. thaliana* were retrieved from BRAD (http://brassicadb.org/brad/). *B. rapa* genes sharing the same *A. thaliana* ortholog were defined as a paralogous set. FDR (false discovery rate) value less than 0.01 and |log_2_(fold change)|≥2 were used to recognize the significance of the gene expression difference. After normalization, hierarchical clustering and k-means clustering analysis of the expression patterns were performed by Mutiexperimental Viewer v4.7 (Saeed et al., 2003).

### GO and KEGG enrichment analysis

To identify putative biological functions and pathways for the DEGs, the Gene Ontology (GO) and Kyoto Encyclopedia of Genes and Genomes (KEGG) database were searched for annotation. The results of GO annotation were submitted to WEGO for GO classification (Ashburner et al., 2000). Enrichment analysis of GO and KEGG was used by the AgriGO and KOBAS2.0 packages, respectively (Du et al., 2010; Xie et al., 2011). GO functional enrichment and KEGG pathway enrichment analysis were also tested at a significance cutoff of 0.05 false discovery rate (FDR).

### Validation of RNA-seq by real time RT-PCR

For the real time quantitative RT-PCR, 1 μg of total RNA was used to synthesize the cDNA using the RevertAid First Strand cDNA Synthesis Kit (Thermo Scientific). Quantitative PCR was performed using the FastStart Universal SYBR Green Master (Roche) according to the manufacturer's instruction on the StepOne Plus Real time PCR Platform (Applied Biosystems). The qRT-PCRs were carried out with the following protocol: 95°C for 10 min, followed by 40 cycles of 95°C for 15 s, and at 60°C for 60 s. The *BrActin* was used as the internal control, which has been shown to be one of the reference genes in Chinese cabbage. After the amplification, the melting curve was determined for specific product. Three independent biological replicates for each sample and three technical replicates for each biological replicate were analyzed. Significant differences of gene expression level between “GHA” and “XK” were evaluated using a student's *t* -test. All of the primers for the selected genes are listed in Table S1.

### Availability of supporting data

The data sets supporting the results of this article are included in the Supplementary Online Materials. The sequencing raw data of this article have been deposited in a SRA database at the NCBI under the accession number: SRP064703.

## Results

### Overview of the RNA sequencing

In total, 10 libraries of Chinese cabbage leaves were created, from plants exposed to normal (CK) and high temperature (37°C) for 1, 6, 12, and 18 h. Totally, 36.8G raw reads were generated (Table 1). After filtering the reads with low quality, the proportion of clean reads were all above 92% for each library. Of the total clean reads from the ten samples, 40.5–43.28% were perfect match, 26.08–28.15% had no more than five base mismatches, 67.24–70.89% were mapped to unique genome locations and 29.11–32.76% were unmapped reads (Table 1). All clean reads were aligned to the reference *B. rapa* genome (v1.5) and a total of 43537 predicted *B. rapa* genes were annotated.

**Table 1:** **Statistic analysis of Chinese cabbage reads in 10 libraries mapped to *B. rapa* reference genome**.

	**Varieties**	**Total reads**	**Perfect Match**	**≥5 Mismatch**	**Unique mapping reads**	**Total Unmapped Reads**
CK	“GHA”	40,950,843	16,868,080 (41.19%)	11,457,693 (27.98%)	28,325,773 (69.17%)	12,625,070 (30.83%)
	“XK”	33,656,232	13,632,073 (40.5%)	8,998,162 (26.74%)	22,630,235 (67.24%)	11,025,997 (32.76%)
1 h	“GHA”	33,340,697	14,250,051 (42.74%)	9,384,813 (28.15%)	23,634,864 (70.89%)	9,705,833 (29.11%)
	“XK”	32,410,775	13,588,176 (41.92%)	9,122,461 (28.15%)	22,710,637 (70.07%)	9,700,138 (29.93%)
6 h	“GHA”	42,468,233	18,241,741 (42.95%)	11,659,028 (27.45%)	29,900,769 (70.41%)	12,567,464 (29.59%)
	“XK”	30,054,440	12,782,217 (42.53%)	8,454,034 (28.13%)	21,236,251 (70.66%)	8,818,189 (29.34%)
12 h	“GHA”	39,689,031	16,738,076 (42.17%)	11,028,057 (27.79%)	27,766,133 (69.96%)	11,922,898 (30.04%)
	“XK”	39,147,008	16,602,499 (42.41%)	10,849,605 (27.72%)	27,452,104 (70.13%)	11,694,904 (29.87%)
18 h	“GHA”	42,069,491	17,802,207 (42.32%)	11,625,527 (27.63%)	29,427,734 (69.95%)	12,641,757 (30.05%)
	“XK”	37,316,428	16,150,331 (43.28%)	9,732,150 (26.08%)	25,882,481 (69.36%)	11,433,947 (30.64%)

### Differential expression profiling of heat treatment between “GHA” and “XK”

**Figure 1:**
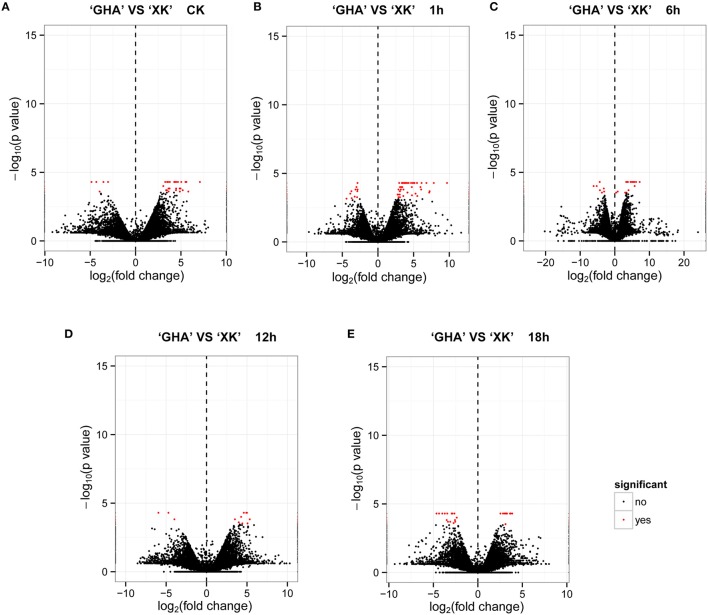
**Volcano plots of the transcriptome between “GHA” and “XK” at different heat treatment stages**. In the volcano plot, statistical significance (log_10_ of *p*-value; Y- axis) has been plotted against log_2_-fold change (X-axis). **(A)** At room temperature (CK). **(B)** 1 h heat treatment. **(C)** 6 h heat treatment. **(D)** 12 h heat treatment. **(E)** 18 h heat treatment.

To investigate the gene expression patterns between heat sensitive variety “GHA” and heat tolerant variety “XK”, FPKM values were calculated for each sample to normalize the expression under different conditions. Volcano plots were constructed to see how many transcripts were significantly regulated during heat treatment for different time periods (Figure 1). The center of the volcano is a line at which fold change is zero, while both sides of the line are indicating down-regulation (negative vales) and up-regulation (positive values), respectively. And the significantly DEGs are represented by red dots with |log_2_ (fold change)|≥2 and FDR value less than 0.001. The results showed that many transcripts were up-regulated in “GHA” at CK and at 1 h after heat treatment (Figures 1A,B). However, more transcripts were up-regulated in “XK” for heat treatment at 18 h (Figure 1E).

**Figure 2:**
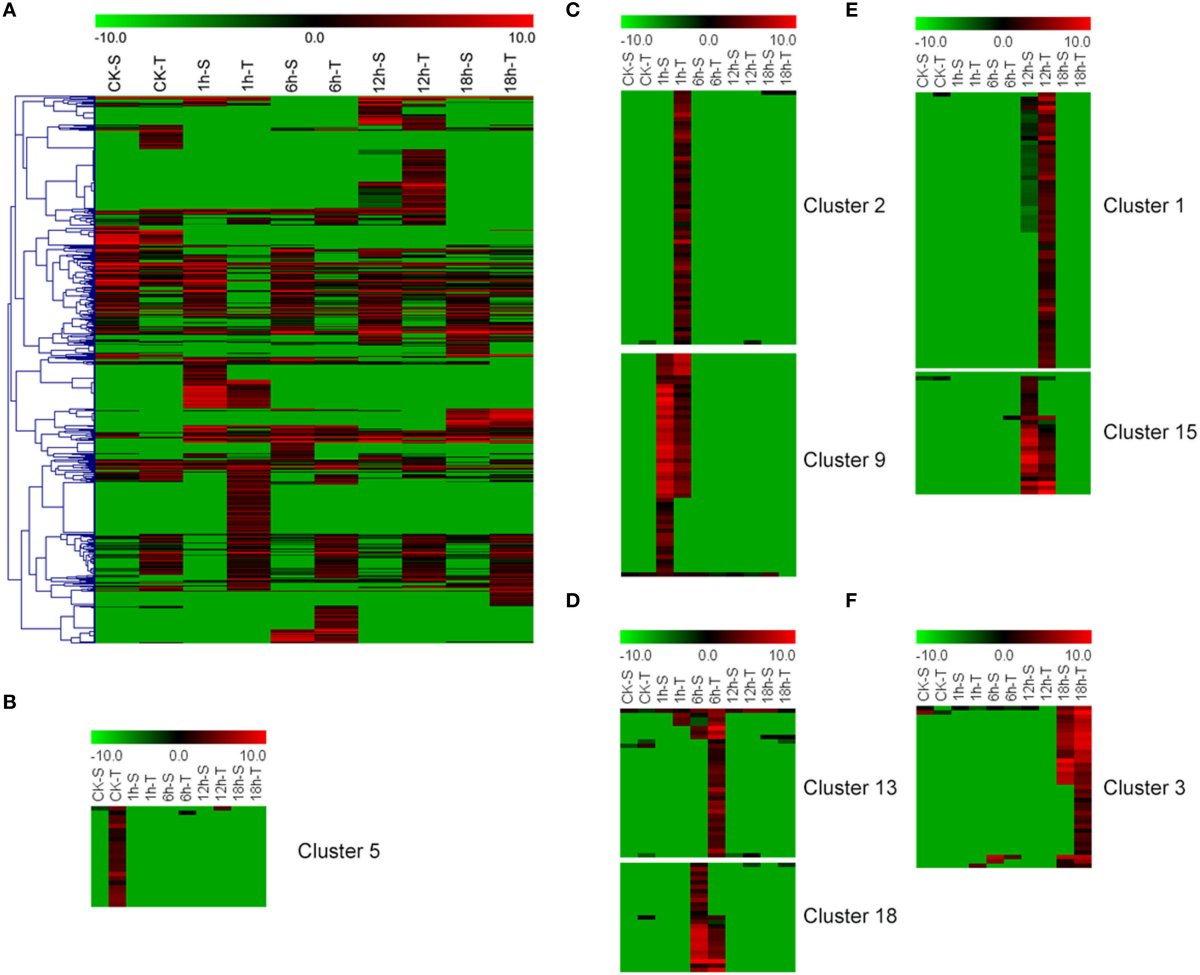
**Heatmap of the differentially expressed genes (DEGs) between “GHA” (S) and “XK” (T) at different heat treatment stages. (A)** Overview of the DEGs under heat stress. **(B)** The specific DEGs at room temperature (CK). **(C)** The specific DEGs at 1 h heat treatment. **(D)** The specific DEGs at 6 h heat treatment. **(E)** The specific DEGs at 12 h heat treatment. **(F)**The specific DEGs at 18 h heat treatment.

A total of 625 DEGs between “GHA” and “XK” were detected by RNA-seq in the present study. Heatmap of these DEGs at all the stages revealed that many DEGs were up-regualted in “XK” (Figure 2A). Clustering analysis showed that large abundant of DEGs were sepcific expressed in each stage (Figures 2B–F, Figure S1). Some of the DEGs were induced in “XK” under room temperature (CK) (Cluster 5) and 1 h heat treatment (Cluster 2) (Figures 2B,C). However, other DEGs of clusters 9, 18, and 15 were highly expressed in “GHA” at 1, 6, and 12 h after heat treatment, respectively (Figures 2C–E).

**Figure 3:**
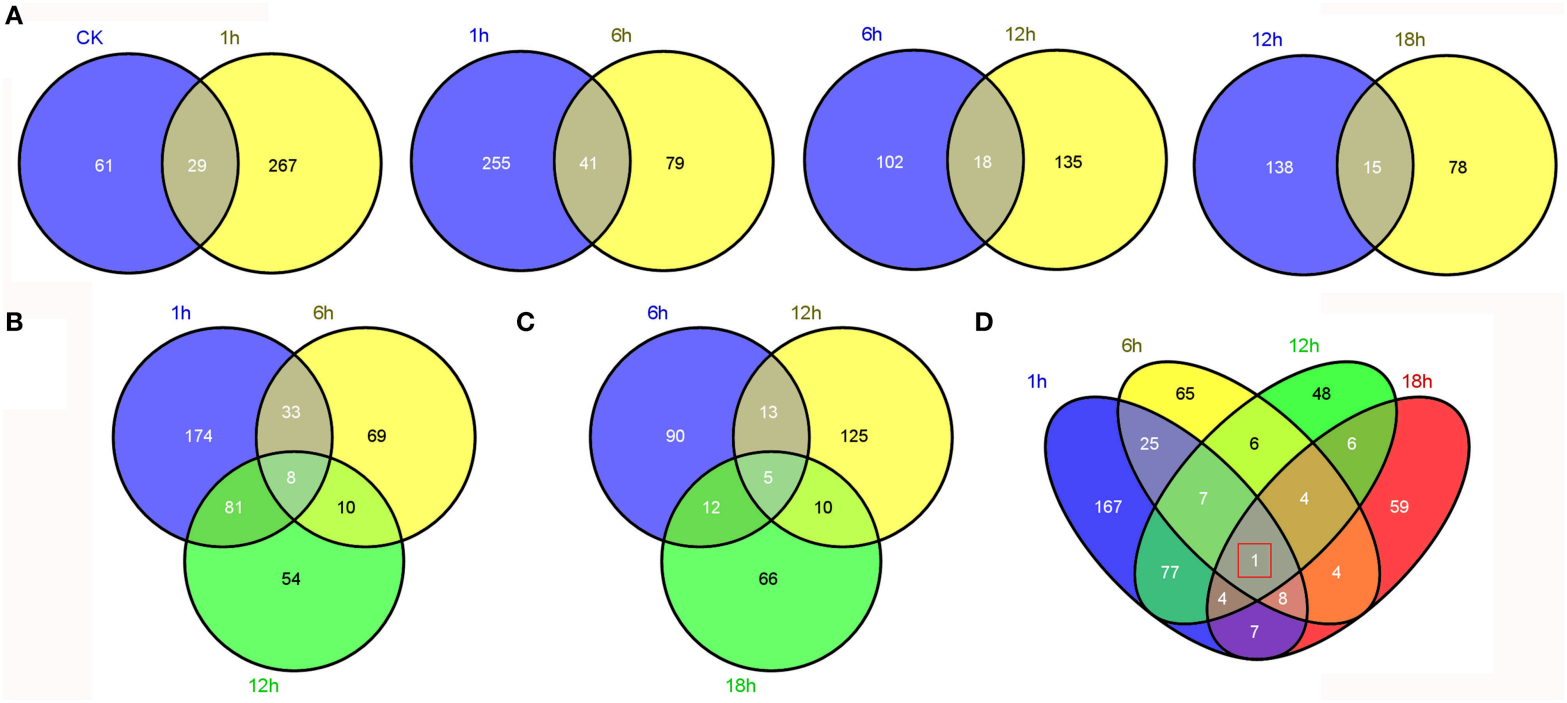
**Comparative analysis of differentially expressed genes (DEGs) between “GHA” and “XK” at different heat treatment stages. (A)** Venn diagram showing the overlap of DEGs between adjacent stages (CK vs. 1 h, 1 vs. 6 h, 6 vs. 12 h, and 12 vs. 18 h). **(B)** Venn diagram showing the overlap of DEGs at 1, 6, and 12 h heat treatment stages. **(C)** Venn diagram showing the overlap of DEGs at 6, 12, and 18 h heat treatment stages. **(D)** Venn diagram showing the overlap of DEGs at 1, 6, 12, and 18 h heat treatment stages.

Comparative transcriptome analysis between “GHA” and “XK” showed that many genes were up or down regulated. Then, Venn diagrams illustrated the number of genes uniquely expressed in each sample or genes that were shared between one or more other samples (Figure 3). A total of 625 genes were differentially expressed in the five samples (Table 2, Table S2). After heat treatment, there were more DEGs with 296, 128, 153 genes at 1, 6, and 12 h than those from other stages, respectively (Table 2). The highest number of DEGs occurred at heat treatment for 1 h. After 1 h of heat stress, 119 and 177 genes were up-regulated and down-regulated in heat-tolerant variety “XK,” respectively (Table 2). Some of the DEGs were shared between the two consecutive stages (Figure 3A). A comparison of the number of DEGs between 1 and 6 h of heat stress showed that 41 DEGs were shared at the two stages (Figure 3A). The number of DEGs simultaneously expressed among 1, 6, and 12 h of heat stress was higher than that expressed simultaneously in the other three successive stages (Figures 3B,C). Moreover, one gene (Bra027596, NAC069) was differentially expressed throughout all the time periods under heat stress (Figure 3D).

**Table 2:** **Differentially expressed genes (DEGs) between varieties “GHA” and “XK” during heat treatment for 1, 6, 12, and 18 h**.

			**Unique in “GHA”**	**Unique in “XK”**
DEGs at CK	Total	90	19	39
	Up-regulated	44		
	Down-regulated	46		
DEGs at 1 h	Total	296	124	108
	Up-regulated	119		
	Down-regulated	177		
DEGs at 6 h	Total	128	30	61
	Up-regulated	68		
	Down-regulated	60		
DEGs at 12 h	Total	153	34	106
	Up-regulated	110		
	Down-regulated	43		
DEGs at 18 h	Total	93	22	44
	Up-regulated	58		
	Down-regulated	43		

### Functional annotation of the DEGs

**Figure 4:**
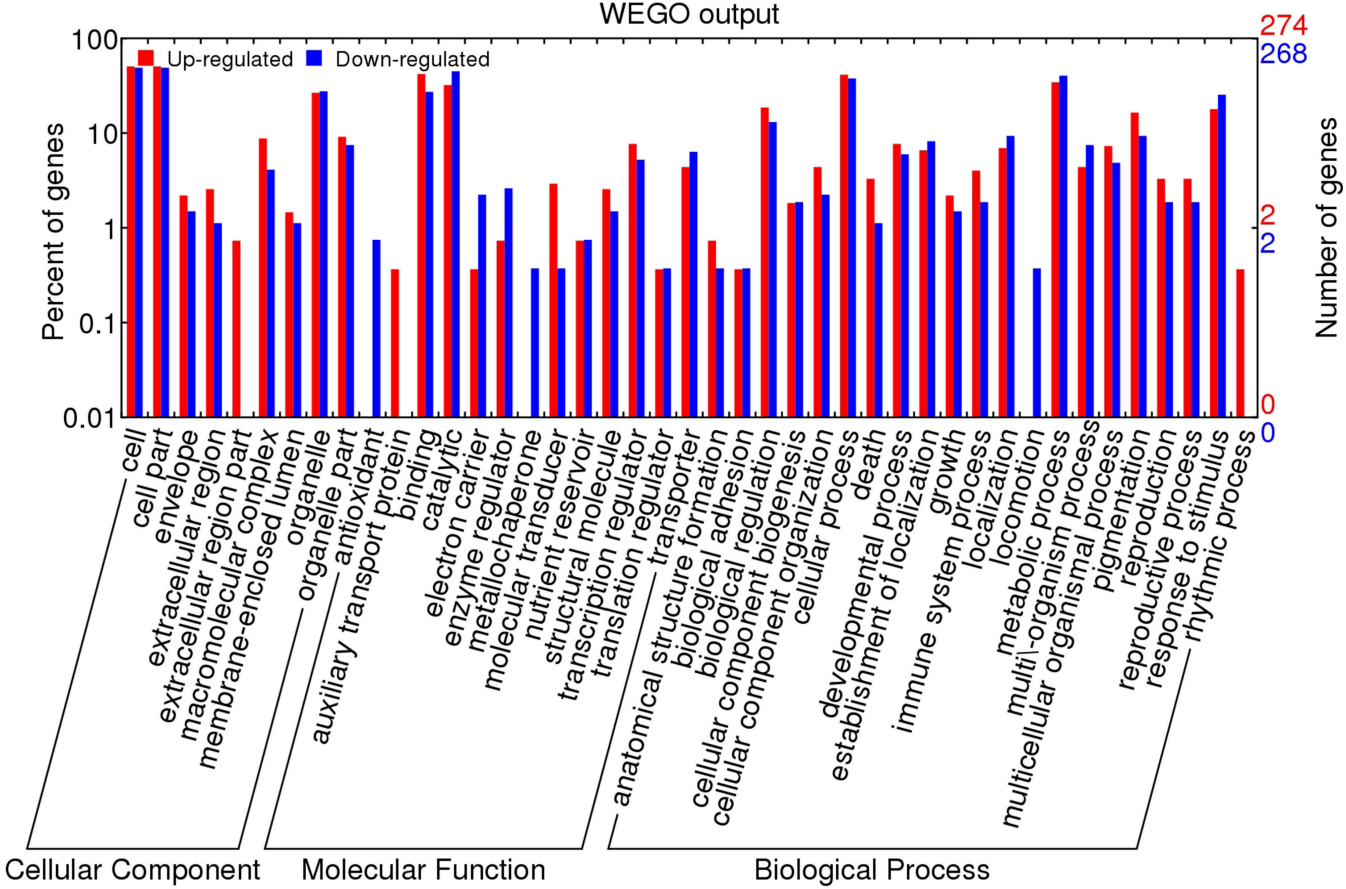
**GO analysis of upregulated and downregulated genes during heat treatment**. The results are divided into three main categories: molecular function, biological process and cellular component. The right Y-axis indicates the number of genes in up-regulated and down-regulated categories. The left Y-axis indicates the percentage of a specific category of genes in that main category.

To functionally annotate the DEGs of heat-sensitive “GHA” and heat-tolerant “XK” varieties, we aligned all of the DEGs against the Gene Ontology (GO) (Ashburner et al., 2000) and Kyoto Encyclopedia of Genes and Genomes (KEGG) (Kanehisa et al., 2008) database. For GO analysis, we annotated genes to three major GO categories: Cell Component (CC), Molecular Function (MF), and Biological Process (BP). The entire set of 625 DEGs was subjected to GO analysis and the summary of detailed annotations for these categories was depicted in Figure 4 by WEGO application (Ye et al., 2006). In the CC category, the terms of “cell” (GO:0005623) and “cell part” (GO:0044464) were the enriched components. “Binding” (GO:0005488) and “catalytic activity” (GO:0003824) were the top two MF terms. The most enriched components of the BP category were “cellular process” (GO:0009987), “metabolic process” (GO:0008152) and “response to stimulus” (GO:0009628).

**Figure 5:**
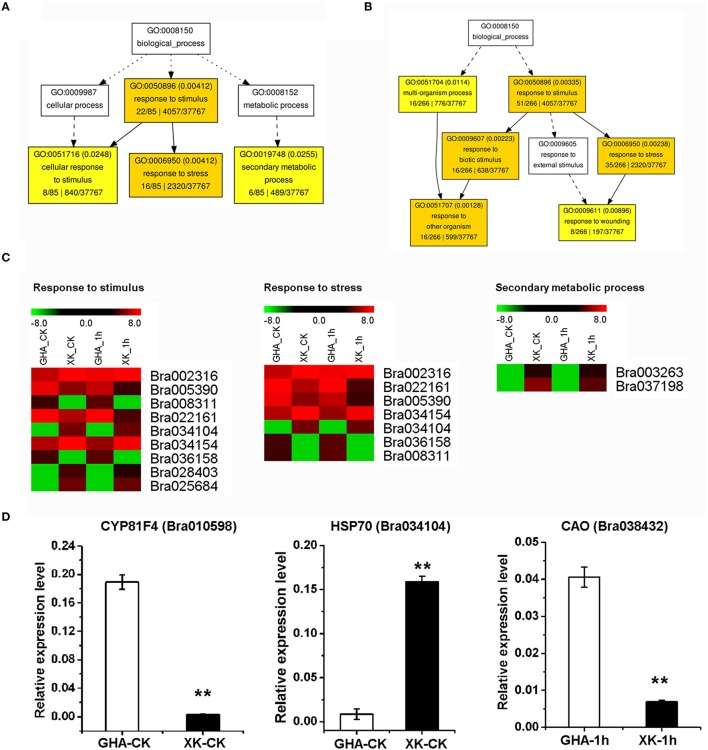
**GO enrichment of differentially expressed genes (DEGs) between “GHA” and “XK” as well as heatmaps of DEGs at CK and 1 h heat treatment. (A)** GO enrichment of DEGs at room temperature (CK). **(B)** GO enrichment of DEGs at 1 h heat treatment. **(C)** Heatmaps of DEGs of enriched GO terms at CK and 1 h heat treatment. **(D)** qRT-PCR of various DEGs between “GHA” and “XK” at CK and 1 h heat treatment. Bra010598, CYP81F4; Bra034104, HSP70 (heat shock protein 70); Bra038432, CAO (copper amine oxidase). The asterisks indicate significant differences between “GHA” and “XK,” as determined by Student's *t*-test (^**^P < 0.01).

**Figure 6:**
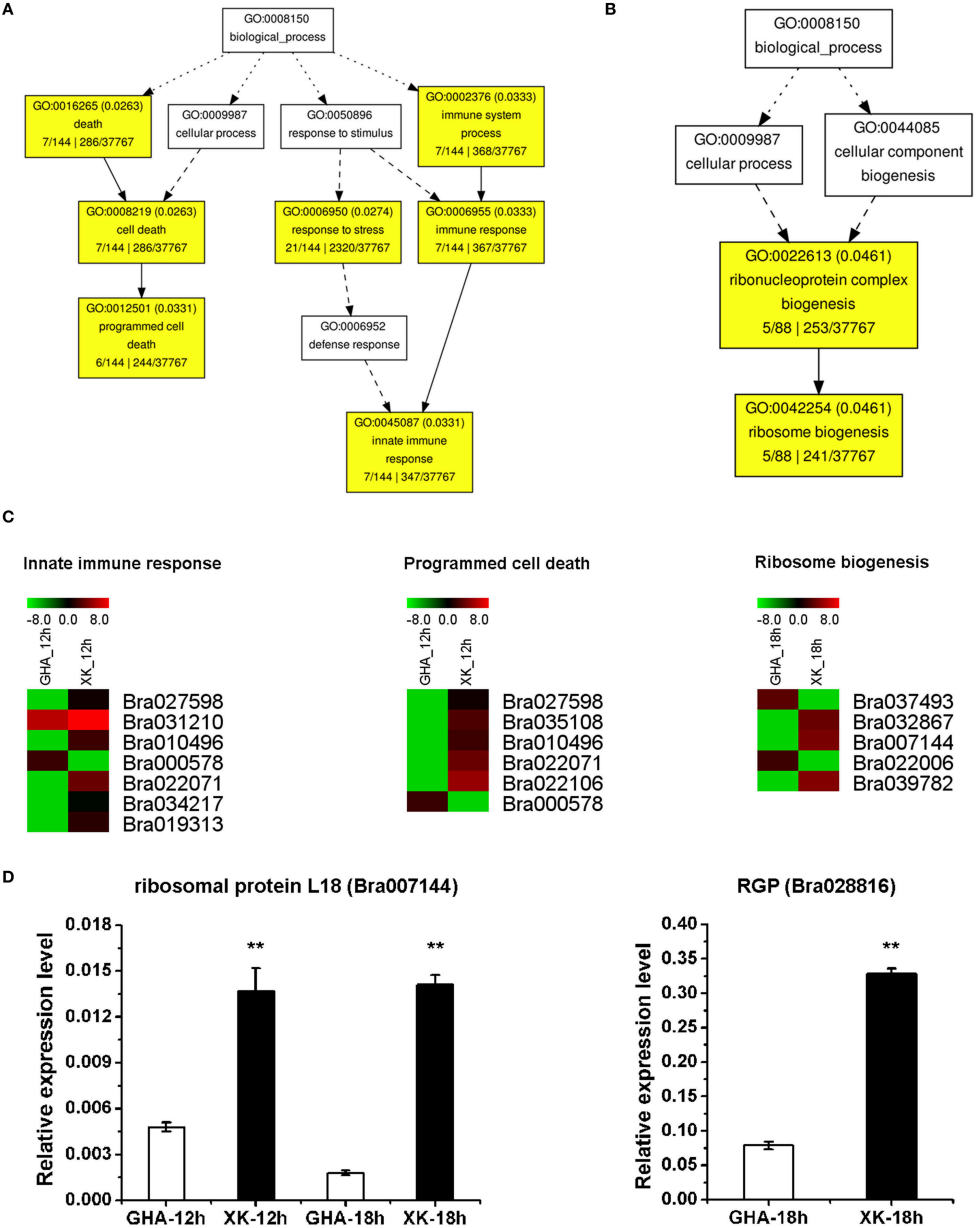
**GO enrichment of differentially expressed genes (DEGs) between “GHA” and “XK” as well as heatmaps of DEGs at 12 and 18 h heat treatment. (A)** GO enrichment of DEGs at 12 heat treatment. **(B)** GO enrichment of DEGs at 18 h heat treatment. **(C)** Heatmaps of DEGs of enriched GO terms at 12 and 18 h heat treatment. **(D)** qRT-PCR analysis of various DEGs between “GHA” and “XK” at 12 and 18 h heat treatment. Bra007144, ribosomal protein L18; Bra028816, RGP (hydroxyproline-rich glycoprotein family protein). The asterisks indicate significant differences between “GHA” and “XK,” as determined by Student's *t*-test (^**^P < 0.01).

During the initial period of heat stress (CK, 1 and 6 h), most DEGs were enriched in the BP category of “response to stimulus” (Figures 5A,B, Table 3, and Tables S3, S4). After heat stress for 12 h, many DEGs were involved in “programmed cell death” or “innate immune response” process (Figure 6A, Tables S3, S4). Then, DEGs of “ribosome biogenesis” was enriched under heat stress for 18 h (Figure 6B, Tables S3, S4).

**Table 3:** **GO analysis (Biological process) results of DEGs between Varieties “GHA” and “XK” during heat treatment for different hours**.

**GO ID**	**Description**	**CK**	**1h**	**6h**	**12h**	**18h**
GO:0050896	Response to stimulus	0.0041^**^	0.0034^**^	0.12	0.056	0.32
GO:0006950	Response to stress	0.0041^**^	0.0024^**^	0.16	0.027^*^	0.25
GO:0019748	Secondary metabolic process	0.00089^**^	3.2e-06^**^	–	–	–
GO:0045087	Innate immune response				0.033^*^	
GO:0012501	Programmed cell death				0.033^*^	
GO:0042254	Ribosome biogenesis					0.046^*^

* FDR ≥ 0.05,

** FDR ≥ 0.01.

KEGG analysis suggested that 294 networks were involved in heat response, including energy metabolism, carbon metabolism, starch and sucrose metabolism, photosynthesis and others (Table S5). The top 10 metabolic pathways possibly regulated by heat stress are presented in Table 4.

**Table 4:** **List of top 10 pathways of DEGs between Varieties “GHA” and “XK”**.

**KEGG ID**	**Pathway**	**Number of transcripts**
ko01200	Carbon metabolism	17
ko00630	Glyoxylate and dicarboxylate metabolism	12
ko00500	Starch and sucrose metabolism	9
ko00940	Phenylpropanoid biosynthesis	9
ko00480	Glutathione metabolism	8
ko05203	Viral carcinogenesis	7
ko05204	Chemical carcinogenesis	7
ko00945	Stilbenoid, diarylheptanoid and gingerol biosynthesis	7
ko00980	Metabolism of xenobiotics by cytochrome P450	7

### Validation of RNA-seq data by quantitative real-time PCR

**Figure 7:**
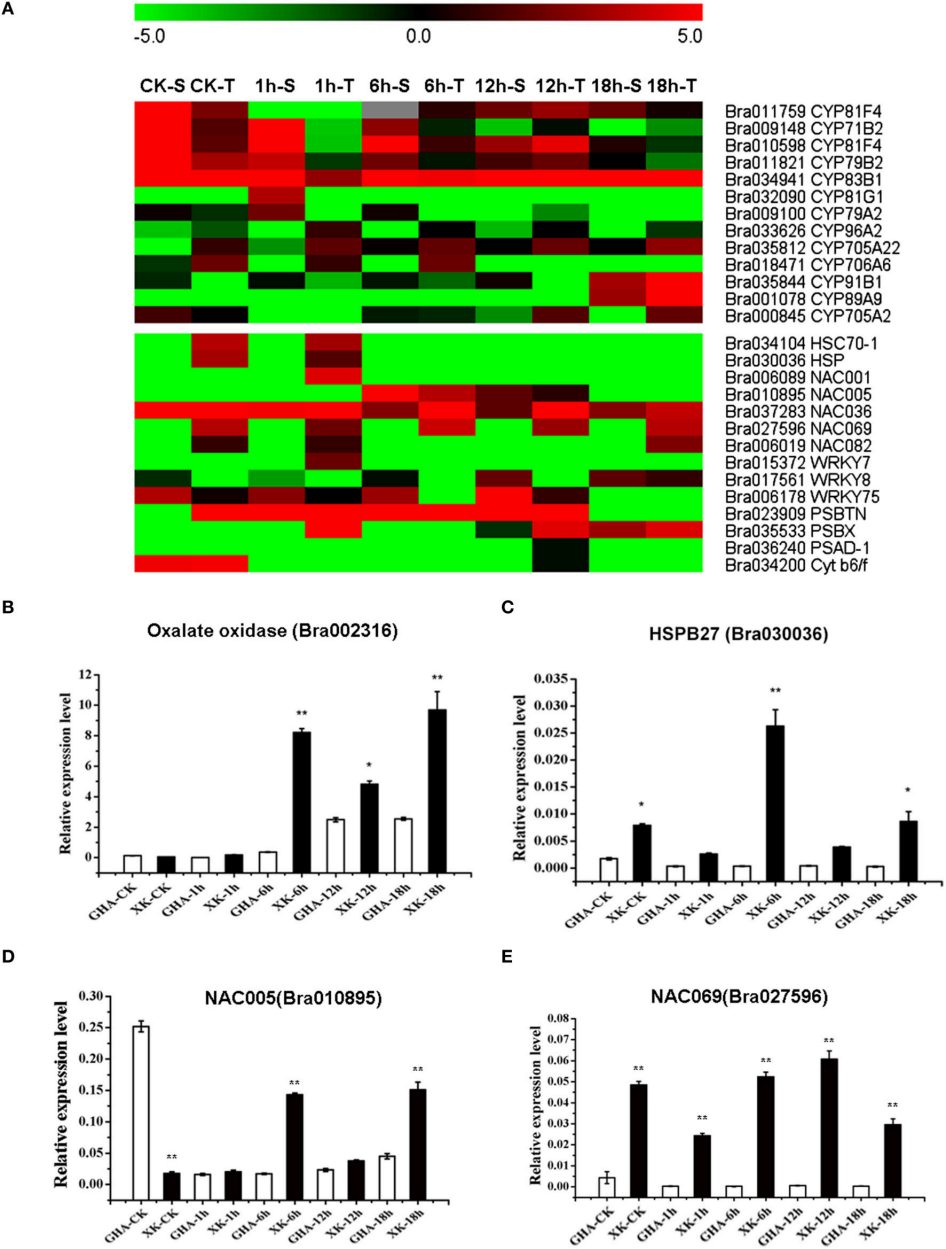
**Heatmaps of differentially expressed genes (DEGs) between “GHA” (S) and “XK” (T) and Validation of the four DEGs from RNA-seq analysis by qRT-PCR analysis. (A)** Heatmap of differentially expressed genes (DEGs) during the heat treatment. **(B–E)** qRT-PCR analysis of four DEGs. **(B)** Bra002316, Oxalate oxidase; **(C)** Bra030036, HSPB27 (heat shock protein binding 27); **(D)** Bra010895, NAC005; **(E)** Bra027596, NAC069. The asterisks indicate significant differences between “GHA” and “XK,” as determined by Student's *t*-test (^**^P < 0.01).

To verify the RNA-seq expression data, we selected nine genes displaying diverse expression profiles during the heat treatment for real-time RT-PCR analysis. The expression levels of five genes were only confirmed at one or two heat treatment stages. Two genes of Bra010598 and Bra038432 were up-regualted in “GHA” at the early heat treatment (CK and 1 h) (Figures 5C,D). However, HSP70 (Bra034104), Bra007144 (rpl18) and Bra028816 (RGP, glycoprotein family protein) were highly expressed in “XK” at 12 or 18 h heat treatment (Figure 5D and Figures 6C,D). These results confirmed the high reliability of the RNA-seq data obtained in the present study. The other four DEGs were further validated for all the heat treatment stages, including NAC005 (Bra010895) and NAC069 (Bra027596), Oxalate oxidase (Bra002316), HSPB27 (Bra030036). As expected, the expression of oxalate oxidase and HSPB27 increased during the heat stress (Figures 7A–C).

### Cytochrome P450 genes associated with heat stress

In higher plants, cytochrome P450s play crucial role in biotic and abiotic stress. Thirteen cytochrome P450 genes were differentially expressed during the heat stress, and most of them were up-regulated in the “GHA” in all the treatments (Figure 7A, Table S4). Five genes (Bra011759, Bra009148, Bra010598, Bra011821, and Bra034941) were highly induced at the early stages of heat stress (CK, 1 and 6 h), while three genes (Bra009148, Bra010598, Bra011821) were down-regulated in “XK” under 1 and 12 h heat stress, respectively (Figure 7A). However, two other genes (Bra035844 and Bra001078) were highly expressed at 18 h of heat stress (Figure 7A). The cytochrome P450 gene CYP96A2 (Bra033626) was especially lowly expressed in “GHA” under CK and 12 h heat stress (Figure 7A, Table S4).

### Genes related to photosynthesis

Photosynthesis is particularly sensitive to heat stress and photosystem II (PSII) with its oxygen- evolving complex (OEC) is the stress-sensitive sites (Allakhverdiev et al., 2008). Two PSII subunit PSBTN (Bra023909) and PSBX (Bra035533) were highly expressed in the heat-tolerant variety “XK” (Figure 7A, Table S4). Moreover, one photosystem I subunit D-1 (Bra036240) was induced in “XK.” Another gene (Bra034200) encoding photosynthetic electron transfer Cytb6/f was also slightly up-regulated in “XK” (Figure 7A, Table S4). The results showed that heat stress affect the photosynthesis in non-heading Chinese cabbage.

### Differentially expression of transcription factors (TF) during the heat stress

HSPs are known to contribute to thermotolerance. We also found two HSPs, Bra034104 (HSP70-1), and Bra030036 (HSP), that were highly expressed in the heat-tolerant variety “XK” during the initial period of heat stress (Figures 7A,C). Some members of the WRKY gene family were reported to be affect by high temperature (Qiu et al., 2004). In this study, one WRKY gene (Bra015372, WRKY7) was highly expressed in “XK” at 1 h of heat treatment. However, two other WRKY genes (Bra017561, WRKY8, and Bra006178, WRKY75) were up-regulated in “GHA” for nearly all the treatments (Figure 7A, Table S2).

NAC proteins are plant-specific TFs which have been shown to be related to plant development and to abiotic and/or biotic stress responses. In this study, one NAC TF (Bra010895, NAC05) was highly induced in “GHA” at the 6 and 18 h of heat treatments, while the other four NAC TFs were up-regulated in “XK” in all the treatments (Figures 7A,D, Table S4). Furthermore, the NAC069 gene was highly expressed in the “XK” at all the stages of heat treatment (Figure 7E).

## Discussion

High temperature influences plant development and can reduce crop yield. To mitigate the effects of heat stress, it is critical to contrive plants that can withstand environmental challenges. In the present study, we investigated the genes that were responsive to high temperature at different stages for the leaves of non-heading Chinese cabbage using RNA-seq. Compared with the transcriptome of a heat-sensitive variety “GHA” and a heat tolerant variety “XK,” potential candidate genes involved in the high temperature response were identified in non-heading Chinese cabbage.

The differential expression analysis of RNA-seq data in “GHA” and “XK” revealed that more DEGs were identified in “GHA” than that of “XK”at 1 h of heat treatment (Figure 1B, Table 2, and Table S2), indicating that “GHA” is more sensitive to heat stress at the early time. However, at the 12 h of heat treatment, many DEGs were up-regulated or specifically expressed in “XK” (Table 2). That may be because many genes involved in heat-tolerance were expressed at this stage in “XK,” such as immune response genes and so on.

Cluster analysis showed that many DEGs were up-regulated in heat tolerant variety “XK” and most of them were involved in “oxidation reduction” or “regulation” process (Figure 2). For example, these genes included oxalate oxidase (Bra002316), HSPB27 (Bra030036), cytochrome P450 (Bra035844, Bra001078, and Bra000845) and NAC069 (Bra027596). These results suggested that oxidative damage was induced during the heat stress and more genes were highly expressed for heat tolerance in non-heading Chinese cabbage.

GO classification analysis showed that the majority of DEGs between “GHA” and “XK” were significantly overrepresented in “response to stimulus,” “secondary metabolic process,” “metabolic process,” “immune response,” “death,” and “ribosome biogenesis” (Figure 4, Table S3). For the DEGs in the four different heat treatment stages, it suggested that the process of responsive to heat stress were divided into three steps. Firstly, GO enrichment analysis revealed that most of the DEGs at the early heat stress stages (1 h) were involved in “response to stimulus” (Figure 5B). These results showed that a number of genes were differentially expressed during the early stage for immediately responding to heat stress. However, at the next time (after 12 h), many genes associated with programmed cell death (PCD) and other immune responsive genes were increased. That may be because the heat stress lead to increased cell death, starting with the PCD and immune system (Figure 6A). At the third step, most of the cell need to be repaired, and many genes involved in ribosome biogenesis were highly expressed at 18 h of heat treatment (Figure 6B).

Cytochromes P450 (P450) is a family of heme-thiolate enzymes involved in the oxidative metabolism of a variety of endogenous and exogenous lipophilic compounds. In this study, many Cyp genes were up-regulated in the heat-sensitive “GHA,” which indicated that much reactive oxygen species (ROS) were generated during the response to heat stress at the early stage (CK, 1 and 6 h; Figure 7A, Table S4). ROS can directly modify cellular macromolecules, leading to a variety of toxic effects, including lipid peroxidation, protein dysfunction, oxidative stress and cell death. GO enrichment analysis also confirmed that a number of DEGs involved in PCD were expressed the 12 h of heat treatment (Figure 6A). DEGs associated with ribosome biogenesis were also found to be expressed at the 18 h of heat treatment (Figure 6B). In rice, transcriptome changes of high night temperature at the early milky stage reported that cytochrome P450 unigene was highly up-regulated in a heat-sensitive rice line (Liao et al., 2015). The results indicated that a higher expression level of the P450 genes would be an index of damage for heat stress.

As signaling molecules, ROS plays an important role in the regulation of abiotic stimuli in plants. ROS include superoxide and hyperoxide, which both contribute to the oxidant state, leading to PCD (Yoshinaga et al., 2005). Oxalate oxidase is involved in the burst of H_2_O_2_ which can induce PCD in the aleurone layer during seedling development (Fath et al., 2000; Lane, 2002). In this study, Mn superoxide dismutase (SOD) (Bra034154) and oxalate oxidase (Bra002316) were up-regulated in “XK” after heat treatment (Figure 7, Table S2). These accumulations of ROS responsive to the heat stress induced PCD associated genes at 12 h of heat treatment (Figure 6A).

Heat stress results in the misfolding of newly synthesized proteins and the denaturation of existing proteins. HSP are responsible for protein folding, and can assist in protein refolding under stress conditions (Wang et al., 2004; Song et al., 2014a). In rice, the interplay between HSP101 and HSA32 can affect thermotolerance in seedlings (Lin et al., 2014). In Arabidopsis and carnation, the expression of heat shock transcription factors (Hsfs) HsfA3 in response to heat stress, has been shown to be dependent on the DREB2A transcription factor (dehydration-responsive element binding protein 2A; Schramm et al., 2008; Wan et al., 2015). In Chinese cabbage NHCC, Hsfs was also showed high connection with DREB2 (Song et al., 2016). In the present study, many HSP genes including HSP70, HSP101 and HSPB27 were induced during the heat stress (Figure 7C, Table S2). Other studies demonstrated that HSP70s play roles in response to heat stress and hydrogen peroxide can enhance ABA-dependent expression of HSP70 to tolerate heat stress (Li et al., 2014; Zhang et al., 2015; Suzuki et al., 2016). Therefore, the up-regulated HSP genes play important role in heat tolerance in non-heading Chinese cabbage.

Many transcription factors are reported to be involved in multiple abiotic stresses including high temperature and cold stress (Bhardwaj et al., 2015; Yang et al., 2015). In this study, we also identified three differentially expressed transcription factors in response to heat stress, including HSF, NAC, and WRKY. The WRKY gene family has been reported to play an important role in plant stress responses. In *A. thaliana*, WRKY25, WRKY26, and WRKY33, were involved in the regulation of resistance to heat stress (Li et al., 2009; Chen et al., 2012). In our study, two WRKY genes were up-regulated in “GHA” for nearly all the treatments and one WRKY gene was highly expressed in “XK” at 1 h of heat treatment (Figure 7A, Table S4). WRKY33 was also reported to be up-regulated in Chinese cabbage Chiifu and may play a role in defense signaling (Dong et al., 2015). These results suggested that WRKY participated in the response to heat stress and played different roles in the damage patterns between the heat-tolerant and heat-sensitive varieties. Previous studies have reported the enhancement of heat stress tolerance in rice by genetic engineering, such as the overexpression of the Arabidopsis molecular chaperone HSP101 or the rice transcription factor OsWRKY11 in transgenic rice. Overexpression of these genes decreased the necrosis of leaves after heat stress treatment in rice plants (Katiyar-Agarwal et al., 2003; Wu et al., 2009).

Most of the NAC transcription factors were also higher expressed in “XK” than in “GHA” (Figure 7A, Table S4). Previous studies suggested that many NAC TFs had been used to improve stress tolerance in crop plants by genetic engineering (Shao et al., 2015). In Arabidopsis, a NAC transcription factor, NAC078 was reported to be responsive to heat stress (Morishita et al., 2009). Over-expression of NAC2 was also reported to increase abiotic stress resistance in rice (Hu et al., 2008). In this study, many NAC TFs were highly expressed in the heat-tolerant variety “XK,” especially, NAC005 and NAC069 (Figures 7A,D,E). Up-regulation of the NAC transcription factors in “XK” suggested that NACs may participate in heat tolerance in Chinese cabbage. In Arabidopsis, NAC069 (AT4G01550) had been reported to be up-regulated after osmotic stress and 50 μM ABA treatment and in NaCl treated roots (Jiang and Deyholos, 2006; Mishra et al., 2014). In Chinese cabbage, Bra019599 (NAC062/NTL6) was greatly increased upon HS in two inbred lines, Chiifu and Kenshin, suggesting their functions in both cold and HT stress (Dong et al., 2015). Therefore, further transgenic research of NAC069 TF in the non-heading Chinese cabbage will elucidate its function in heat tolerance.

## Conclusion

In this study, we compared the transcriptome of heat-sensitive and heat-tolerant non-heading Chinese cabbage varieties in response to heat stress using RNA-seq. Approximately 625 genes were differentially expressed between the two varieties “GHA” and “XK.” The responsive genes can be divided into three phases according to increasing time periods of heat treatment: response to stimulus, programmed cell death, and ribosome biogenesis. Futhermore, NAC069 was up-regulated in heat-tolerant “XK” at all the heat treatment stages, indicating its roles in heat tolerance. The candidate genes will provide genetic resources for improving the heat-tolerance characteristics in non-heading Chinese cabbage. These results could be also used for future studies of the molecular mechanism of heat stress in plants.

## Author contributions

Designed the experiments: GZ, AW, and JH. Performed the experiments: AW, JH, XH, and XL, Analyzed the data: JH, XL, and ZY. Wrote the paper: AW, JH, and GZ. All authors read and approved the final manuscript.

### Conflict of interest statement

The authors declare that the research was conducted in the absence of any commercial or financial relationships that could be construed as a potential conflict of interest.

## Supplementary material

Figure S1 **All the expression patterns of the 625 DEGs between “GHA” and “XK” at different stages of heat treatment by clustering analysis in non-heading Chinese cabbage**.

Table S1 **Primers used in the study for quantitative RT-PCR**.

Table S2 **Differentially expressed genes between “GHA” and “XK” at different heat treatment stages**.

Table S3 **GO enrichment of differentially expressed genes at different heat treatment stages**.

Table S4 **Differentially expressed genes of GO enrichment terms during the heat treatment**.

Table S5 **KEGG pathway of differentially expressed genes at different heat treatment stages**.
