# Supplementary figures and images for: Comparative Transcriptome Analysis Reveals Heat-Responsive Genes in Chinese Cabbage (Brassica rapa ssp. chinensis)
Source: Front Plant Sci. 2016 Jun 28;7:939. doi: 10.3389/fpls.2016.00939 (PMC4923122; doi:10.3389/fpls.2016.00939)

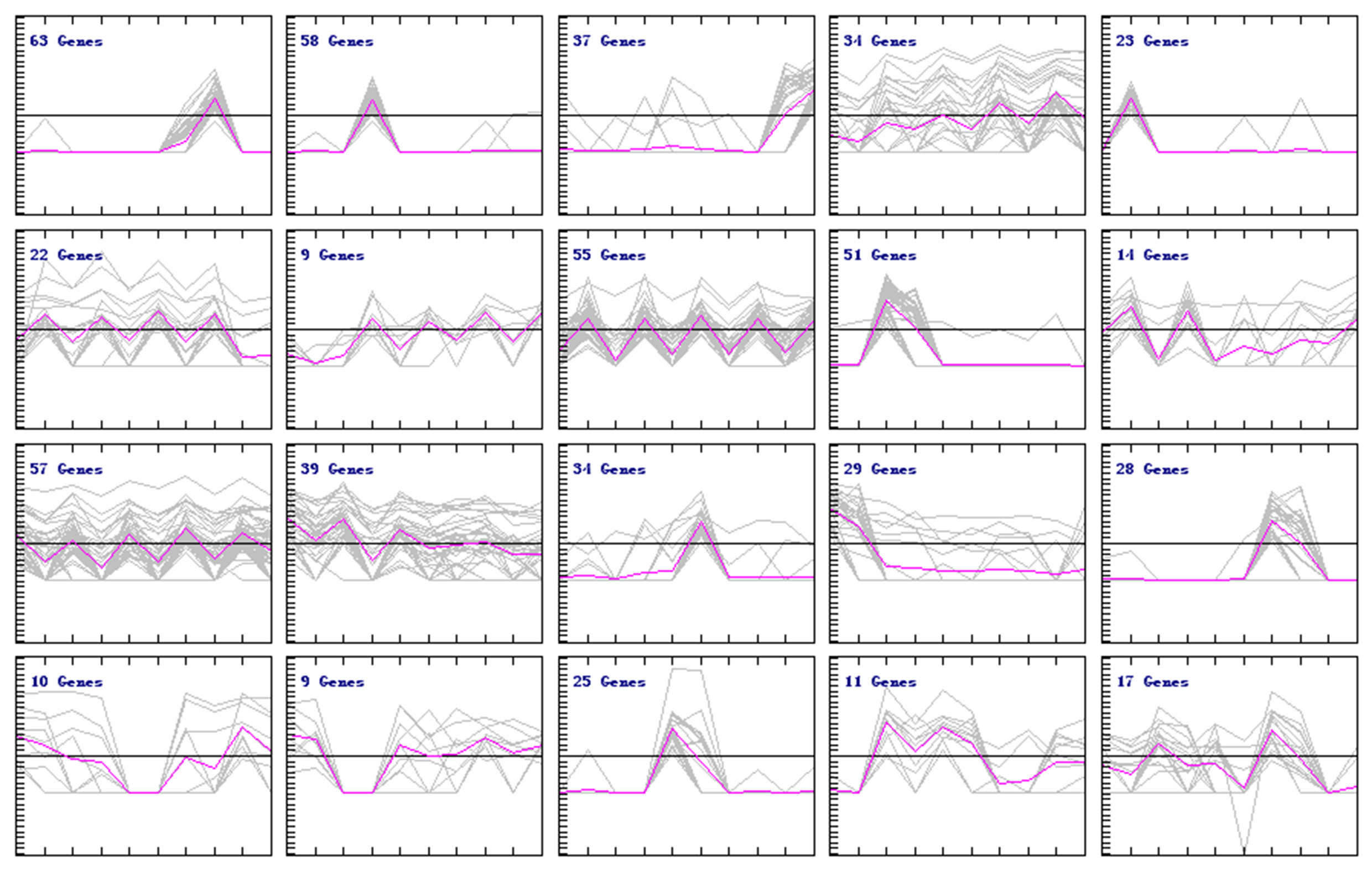

Supplement: Figure S1 — All the expression patterns of the 625 DEGs between “GHA” and “XK” at different stages of heat treatment by clustering analysis in non-heading Chinese cabbage. [file Image1.TIFF]
